# Extra of the Western Journal of Medicine and Surgery

**Published:** 1851-12

**Authors:** 


### EXTRA

OF THE

### WESTERN JOURNAL OF MEDICINE

### AND SURGERY.

---

Dr. Bullitt having prepared an extra edition of his new batch of charges upon me, I republish my response, contained in the July number of the Western Journal of Medicine and Surgery, to his gratuitous and unprovoked assault made upon Dr. Lewis Rogers, Dr. Lyle and myself, in the June number of the Transylvania Journal of Medicine. Neither of us had injured Dr. Bullitt in any way—we had not alluded to him, nor attempted to provoke him. Yet, it is questionable whether the records of medicine contain anything more obnoxious to the laws of the land, to medical ethics, or to sound morals, than Dr. Bullitt's attack. It is true he singles me out for his aspersions, but this only adds to the dark hues of his case. He levels his maledictions at the treatment of a case of accidental disease, in which Dr. Rogers, Dr. Lyle and myself were in constant attendance, but represents that I was the only physician in the case. If a medical editor can thus arraign private practitioners upon the pages of his journal, and open upon them the sluices of malignity and slander, it is time the profession knew it. If medical journals are thus to be diverted from science, and converted into Police Gazettes, every practitioner of medicine should prepare to guard himself, and protect the rights and privileges of the profession. But I am persuaded that neither professional nor public sentiment will sanction such violence, outrage, and iniquity.

The crimes of slander and calumny pollute the atmosphere of society, and destroy the sources of happiness. He that loves to deal in these things, and who makes them the object of his continual love and study, is unsafe as a companion, and cannot be trustworthy in anything. There is no excellence too high for his poisonous arrows, there is nothing too low for his grovelling propensities. No purity in life, no amount of integrity, no care nor prudence is a panoply of sufficient protection from the venom of slander.

> "No might nor greatness in mortality
> Can censure 'scape: back-wounding calumny
> The whitest virtue strikes. What king so strong,
> Can tie the gall up in the slanderous tongue?"

The unhappy feuds of medical men are calculated to injure not only those directly engaged in them, but the whole profession. The public mind becomes surfeited with the bitter denunciations and criminations, and draws no distinction between the innocent and guilty, leaves the regular walks of the profession, and encourages charlatans and quacks. It is the imperative duty of all to put a speedy end to these quarrels, in order to guard the best interests of the profession.

A very cursory examination of Dr. Bullitt's article was sufficient to reveal its full character. It was accepted as a gratuitous, unprovoked series of insults, and was treated as such in the republication I have made from the July No. of the Western Journal of Medicine. How long could a Journal of Medicine stand, devoted to such pursuits as these? How long would the instincts of the profession permit a Journal to live, that would embark in the business of hunting up the gossip of private practice to spread before the readers of scientific Journals? The law of self-preservation should prompt every professional man to shun such practices. But if it is right and proper in one case, it would be in all. This affair of mine then, is not exclusively a personal one—the whole profession is involved in it, and should make common cause against the Ishmael who has commenced running this muck.

I appeal to the profession both in the city and throughout the country to examine with care the response I made to the libels of the June number of the Transylvania Journal. That it was severe I know, but let any physician put himself in my place and ask himself what his feelings would have been under such a great provocation. We have laws to regulate all these matters, and if any improper conduct had been pursued, I was amenable to the laws of the profession, not to Dr. Bullitt. And when he stepped aside from his proper sphere to make a slanderous attack upon three physicians, in their private practice, I hurled back upon him his insults and outrages.

Dr. Bullitt is so well convinced of his inability to make any defence of his conduct in this particular, that he attempts to hide it in a corner, while he splutters largely about other things. He hopes to draw my attention and the attention of his readers away from an outrage that admits of no apology or excuse, while he belabors something else. But this "wriggling lubricity" shall not avail him. "The cords of his sins shall hold him," until he admits his wrong, or quietly confesses judgment.

### CASE OF SULPHURETTED HYDROGEN GAS POISONING.

BY THEODORE S. BELL, M. D.

---

In the May number of the Western Journal of Medicine and Surgery, I reported a case of poisoning by Sulphuretted Hydrogen Gas, and endeavored to give as faithful a picture of the case under review, as a sincere desire to be faithful and accurate would enable me to give. The report of that case has proved to be the groundwork of one of the most scurrilous and vituperative assaults it has ever been my lot to read. Every principle of professional decency and courtesy was thrown aside in the article referred to; misrepresentation and error reigned supreme, the most absurd and ridiculous medical statements and opinions were uttered with as much gravity as though they had been tested by long experience, and were among the foundation stones of practical medicine. It is a matter of some importance that a portion of this extraordinary exhibition of Medical courtesy and liberality shall be examined and fairly tested, in order that we may find out what is the truth, and to whom its utterance belongs. The truth of my report shall be thoroughly established.

Before entering, however, upon this examination, I must express my obligations to Dr. Henry M. Bullitt, the author of the precious morceau in question, for the opportunity he has given me for doing justice to the long cherished regard I have felt for Dr. Powell, since the commencement of a friendship of many years' duration.

There is no gentleman in the profession for whom I feel more regard, nor one for whose medical opinions I entertain more respect. A professional intercourse with

Dr. Powell, of many years' existence has proved one of the most pleasant of my medical career, and entertaining such feelings as these, it would be a singular thing for me to select Dr. Powell as the object of public insult, and grossly improper treatment. The act would have been without a motive, and could have plead no excuse. A recent number of the Transylvania Journal has made a charge of this kind of conduct, the text of a singularly ludicrous homily on medical ethics, in which a violation of the practical part of all medical courtesy, largely predominates over the sermonizing. The accusation is based upon the remarks, in the report alluded to, which I made upon the use of water as an agent for the destruction of sulphuretted hydrogen gas. At the time those remarks were made, so far from having any knowledge that the use of water was in obedience to professional advice, I had strong reasons for believing that the water was used *against* medical advice. The only history I had of the matter, on the night of the disaster, was from Dr. C. L. Lyle, one of the witnesses paraded before the readers of the Transylvania Journal. Dr. Lyle stated to me that he was present when the water was used, and that he opposed its use while the victims were in the pit; that he entreated the people to stop its use, but, he said, they seemed to be wild, and poured the water in torrents. This was all the reliable testimony before me when my criticism was made, and I rested upon its faith. That testimony said very plainly that science opposed a popular fallacy, and I knew no better, until Dr. Powell himself informed me of his connection with the use of the water. Of Dr. Fry's agency I never heard until I saw it announced in the Transylvania Journal. I owe this explanation to Drs. Powell and Fry, and assure them that had I known of their connection with the order for the use of the water, my criticism would have been of a different character. I should have contented myself with the expression of a preference for the use of fire rather than water. One of the last things I should have done, would have been to commit a wanton, unprovoked and motiveless attack upon the reputation of two gentlemen who possess a large share of my esteem and regard.

I should have preferred dropping this part of the subject with this *amende* to Drs. Powell and Fry, but Dr. Bullitt's course forbids the excercise of such a preference. Without undertaking to affirm or deny my former remarks on the use of water in the destruction of sulphuretted hydrogen gas, I shall examine Dr. Bullitt's chemistry, physiology, toxicology, and therapeutics, for the Dr. has contrived to make quite a lively display on all these points. My erudite volunteer instructer informs my deplorable dullness with the refreshing knowledge that water absorbs sulphuretted hydrogen gas, a fact which I have a vague recollection of having heard before. But Dr. Bullitt instructs me only half way, for he fails to inform me that water parts with sulphuretted hydrogen gas quite as readily as it absorbs it. By way of reciprocity, I would inform Dr. Bullitt that water can be poured into a seive, but my impression is that it will not tarry long in that utensil. If Dr. Bullitt had earnestly pressed his surprising and laborious investigations, he would have found that Sir M. Brunel was much annoyed by sulphuretted hydrogen gas in the construction of the Thames tunnel. The workmen sickened, and a number of them died. The subject was referred to an accomplished toxicologist, and upon examination he found that the gas escaped from the action of the river on iron pyrites, and the large volume of the river Thames was not able to absorb and hold the gas. It continued to annoy the workmen for months, shooting out upon them at unexpected times. Now with such a well known fact as this before my mind, am I to believe Dr. Bullitt's gratuitous assertions, and unsustained speculations? I differ from the high authority of Dr.

Bullitt, with great deference, but even Aristotle was not infallible. Dr. Bullitt should have sustained himself with authorities, and closed the question upon me, and he will no doubt do it—when he can find them. Instead of taunting me with my ignorance of the "authorities" it would have been commendable in Dr. Bullitt to have shown some acquaintance with them himself. If he can find one "authority" in toxicology who gives any kind of countenance to his views of the "physiology of respiration;" if he will produce one who even leans towards an approbation of his notion that the best thing to be done for a patient, who is immersed in a gas that has already "partially or entirely suspended respiration." is to force him to respire this deadly gas again, I will surrender at discretion. Now let the erudite Dr. Bullitt wade among the tomes. I have made him a fair proposition, and will abide by its terms. There is no escape for Dr. Bullitt—he must either find "authorities" for his notions of exciting persons to the breathing of sulphuretted hydrogen gas, or he must admit that his indecent references to my ignorance were sadly out of joint as coming from him.

Dr. Bullitt says that it is entirely proper, when patients are in an atmosphere of gas, "so irrespirable as to either partially or entirely suspend respiration," to force them to breath it again. If so, why be in a hurry to get the patients out of the gas? why not play on the "physiolgy of respiration" with water, until the patients recover? Dr. Bullitt would have us believe that in proportion to the baleful effects of the gas, must be the effort of the physician to make the patient breathe more. According to Dr. Bullit in a case where respiration is only partially suspended, the patient may be safely left to gasp at the delightful gas, but when the poison has entirely suspended respiration, efforts must be made to arouse the patient to renewed struggles for more of the gas! For my part, probably for want of what my "general knowledge did not supply me," I would not desire a healthy man to breathe sulphuretted hydrogen gas, and I would not try to force a man to do it, who had already got a sufficient quantity in him to suspend respiration. Dr. Bullitt may consider me very stupid in not being convinced by his intellectual processes, but I cannot help it—I will not believe that any human being should be made to breathe sulphuretted hydrogen gas. I did all I could to submit my mind to the guidance of Dr. Bullitt, because I know the exceeding value of his medical opinions.

But let us proceed :

Dr. Bullitt says: "these men" (those in the pit) "were not in an atmosphere of absolutely irrespirable gas, but by the inhalation of sulphuretted hydrogen, respiration had become partially or entirely suspended, in consequence of the impression of the poison upon the nervous centers." This is Dr. Bullitt's reasoning, not Dr. Powell's nor Dr. Fry's. It is with Dr. Bullitt's presentation of the argument that I am now concerned, and as we are in the hands of a great master of physiology, beggars may pick up nutritive crumbs from his well stored table. I think myself very fortunate in receiving gratuitious instruction from Dr. Bullitt in chemistry and physiology, and the reader will find the extent of my obligations when I get through with this investigation.

Dr. Bullitt asserts that "respiration was suspended in consequence of the impression of the poison upon the nervous centers." Such famous reasoners as Dr. Bullitt are not expected to prove anything, but I should like to know what proof there is for the above reckless assertion. The statement, however, shows how ignorant Dr. Bullitt is of the subject on which he attempted to write. The attention of Dr. Bullitt was directed to the "physiology of respiration," and in the whole of the assault upon me, the entire coterie were so taken up with the respiratory movements, that there is not a hint thrown in all their learned writings that sulphuretted hydrogen gas can be fatal in any other way than by the lungs. This one fact shows how little any of the writing champions knew of what they were writing about. They expect the profession to believe them when they say that patients immersed in sulphuretted hydrogen gas, are properly treated if the respiratory functions can be forced to act. *But sulphuretted hydrogen gas is just as fatal to human life when acting upon the skin, as when breathed,* a fact stated by all the principal toxicologists. If a man were immersed to his neck in sulphuretted hydrogen gas, and his breathing organs were protected so as to have nothing to breathe but atmospheric air, the gas would as certainly kill him, as though he breathed it. What then becomes of all of Dr. Bullitt's twaddle about nervous centers, physiology of respiration, &c.? I feel exceedingly happy in the fact that I am not a professor of general physiology, for the profession might expect me to understand some of the *particulars* of my department. They would at least expect me not to introduce Hahneman's *similia similibus curantur* into a pit filled with smlphuretted hydrogen gas, as regular scientific treatment, as Dr. Bullitt does, when *he* proposes to cure persons in whom respiration is entirely suspended by sulphuretted hydrogen gas, by making the victims breathe more of the gas.

Such raw material may do to hash up for very green students, but no intelligent mind will ever swallow it with any expectation that assimilation will take place. The man who has to feed largely on such stuff is to be pitied.

On Dr. Bullitt's physiological principles, there was no necessity for any haste in getting the victims out of the pit, for water would rouse them to breathing, and that was enough. But I am informed that Drs. Powell and Fry made the most commendable efforts to remove the patients as soon as possible, and to that activity the victims are mainly indebted for their lives. Dr. Bullitt has such confused notions of the subject, that he endorses the statement that Mr. A—— was saved by the presence of mind that enabled him *not* to breathe the gas, and declares that the victims in the same pit, where Mr. A—— saved his life by not breathing, were saved by being made to breathe a gas that was so little irrespirable as to be able only to suspend respiration entirely. And this is my gratuitous, volunteer instructor, who dispenses knowledge that I can safely and prosperously dispense with!

But I am not done with my volunteer instructor. After Dr. Bullitt's sneer at my non-consultation of authorities, our readers may be interested in seeing a specimen of Dr. Bullitt's understanding of authorities. The Doctor taunts me with my ignorance, and in order to prove that it is proper to make people breathe irrespirable gas by pouring water upon them, quotes the following from Orfila: "*Exposure of the patient to the open air*, *aspersions* with cold vinegar and water, friction with a strong hair brush. These form the first assistence to be given to persons afflicted by privies." I cannot see what assistance Dr. Bullitt gets from this statement of Orfila's judgment. It condemns Dr. Bullitt's entire views of the treatment, and sustains the opinions advanced in my report. The very first thing is to get *the patient into the open air*, *away from the gas*, and then cold water or vinegar is to be *sprinkled* over the sufferers. Is this what Dr. Bullitt advocates when he talks about making the patients respire the noxious gas, by *affusions* of cold water? If I could make no better use of Orfila than this, I should let him rest in quiet.

Once more on the "authorities!" In my former notice I intended to show that fire was a better means for ridding a pit of sulphuretted hydrogen gas, than water, and Dr. Bullitt grows indignant on the subject, complains of my ignorance, and maks an admirable display of his own.—With what kind of countenance can Dr. Bullitt look at the following quotation, from the most recent writer on medical jurisprudence, one of the best and highest of the authorities? Taylor says: "Tne best plan for getting rid of the gas is by a free exposure of the locality, *or by exciting active combustion in it*." The latter recommendation could easily have been accomplished without injuring the persons in the pit. Dr. Bullitt must hunt up his authorities, and he must find them, or occupy a very unenviable position. I give him the run of Guy, Apjohn, Christison, Parent Duchatelet, Taylor and Michael Lèvy, and if he can find anything in them to sustain him, I yield every thing. But he must remember that I never objected to the *aspertion* of cold water on persons poisoned by the sulphuretted hydrogen gas, *after they were removed to a pure atmosphere.* If Dr. Bullitt were to see a drowning man, gasping for atmospheric air, under water, he would keep the man in the water because that fluid rouses "the physiology of respiration," and make the gasping man breathe more water, that fluid not being absolutely irrespirable.

This would not be my course, and this constitutes the difference between Dr. Bullitt and myself. But the Docter is a Professor of Physiology and I am not, and that may make a species of compensation or balance.

If Dr. Bullitt has really discovered a new method of treating patients poisoned with sulphuretted hydrogen gas; if he stands in direct opposition to every authority known in toxicology, and is so deeply imbued with fresh knowledge on the "physiology of respiration," as to be able to assert that the proper method of treating patients almost deprived of life by breathing a fatal gas is to make them breathe more of the noxious agent; if he is well established in the facts that the respiratory functions alone are to be attended to, and that the toxicologists are wrong in declaring that the action of the gas is just as fatal when applied to the skin, as when it is breathed: if Dr. Bullitt is firm in all these novelties, he owes it to the profession to establish the doctrine. A field of glory and honor is before him; when he accomplishes his mission, he will be hailed as one of the great benefactors of his species, and the flood-tide of fame awaits him. Even I, humble as my merits are, will join in weaving a chaplet of laurel for his honored brow, and I may at least gather the glory of having appreciated his merits.

It would be unreasonable to suppose that after such a spreading of all the glories of intellection, as I have recorded, Dr. Bullitt would have shown some signs of exhaustion, but he has marvellous gifts of continuance. He endorses the following extraordinary statement:

"We were much gratified in a few minutes by observing that he" (Mr. F——) "began to breathe freer, his pulse at the wrist could be felt, and it seemed to get stronger and stronger, after each inspiration; at the expiration of fifteen or twenty minutes, Drs. Lyle, Fry, and myself considered Mr. F—— in a fair way of recovery; his breathing was now full and regular, but slightly stertorous, his pulse both as to volume and frequency perfectly natural; there still remained, however, a profound coma; our only apprehension was the effects of the venous congestion of the brain, and we therefore determined to bleed him from the arm."

It is difficult to characterise this statement in terms that are at once suitable and becoming, but there is scarcely the shadow of truth in it. The attempt to connect Dr. Lyle with the statement is atrocious. That gentleman was an active, faithful, and efficient coadjutor with Dr. Lewis Rogers and myself in the treatment of the case referred to, and the object in trying to use his name in the above connection is too obvious to require comment. Dr. Lyle never authorized his name to be used as confirmatory of the above statement, and he went over the items of it with me, and declared that the "breathing was not full and regular, and the pulse was not "natural, both as to volume and frequency." At the time when these symptoms were said to be present, I was sitting by the side of the patient; there were long intervals when there was no attempt at breathing—all the attempts that were made were spasmodic gaspings after air—my finger was on the wrist almost constantly for five hours, and at no time in all that period did the pulse make any approach to naturalness. During the time the patient laid in the porch, the pulse was often absent, and when present, could scarcely be felt. Just before the removal of the patient from the porch, Dr. Fry asked me respecting the pulse, and after feeling it some time, I replied that it was scarcely perceptible. He manifested no kind of surprise in finding that it had lost its "natural volume and frequency," but on the contrary, looked quite like himself.

I here republish my true statement of the condition of the patient in the porch: "The entire surface of the body was cold and apparently dead—the breathing, if the spasmodic attempts after air that were made by the sufferer can properly be called breathing, seemed to carry no air to the lungs. Mustard had been applied, but it produced no effect, and ammonia to the nose gave no sign of its presence. It would be difficult to exaggerate the gravity of the symptoms. The skin was universally cold, as cold as death itself could make it; the spasmodic attempts to get air, already described; a pulse sometimes manifesting itself in a feeble flutter, and at other times altogether absent, and this state of things persisted in fully for three hours, and to a considerable extent for six hours, constituted one of the most serious forms of imminent danger I have ever seen."

Now it would be difficult to find a much greater difference between two statements, than is found between the one endorsed by Dr. Bullitt and the one I gave. I think that my word is entitled to quite as much credibility as Dr. Bullitt's, and that far we are even. Dr. Bullitt attempts to lean on Dr. Lyle, in making his endorsement, but, Dr. Lyle says, without any authority from him. Dr. Lyle read over with me, since Dr. Bullitt's publication, each item of my description, and affirms that each and every part of it is true, and that the symptoms I described were such as were present. The witness thus relied upon by Dr. Bullitt sustains every statement I made as to the symptoms in the case. He coincides with me in the declaration that the symptoms thus recorded, remained in full existence for three hours, consequently, they were present when Dr. Rogers came in to the case, an hour after I took part in it, and Dr. Rogers sustains my statement in every particular. Dr. Lyle says they were present from the time I reached the case to the time that Dr. Rogers joined us, and Dr. Rogers and Dr. Lyle vouch for the full truth of the report. Dr. Rogers read the report carefully before it was published, and remarked to me that so far as the description of the case and the treatment were concerned, the report was as accurate as it could be. Dr. Lyle has heard the present written statements, and affirms they are correct. Certainly Dr. Lyle, Dr. Rogers, and myself are able to know the condition of a patient, and there was the most full and complete unity of views among us in this case throghout our connection with it. I am willing to rest the matter on the corroborative testimony of Drs. Rogers and Lyle.

What then becomes of the paltry attempt to falsify the statement of my report, and to injure the character of three physicians who were honestly and faithfully performing their professional duties? The witness relied on deserts the conspirators, and is true to his own honorable instincts and to professional justice. And when it is needed, I can multiply proof in support of my description of the case.

But let us return to the physiological endorser. I have sounded the shallows of his chemical and physiological knowledge, and shall now attend to his character as a pathologist. While Dr. Bullitt was endorsing the statements I have quoted, I think he might have inquired into the consistency and reasonableness of his bond. The statement on the face of it is that "the patient was in a fair way of recovery," when it is admitted there was "a profound coma" and "stertorous breathing."—What wonderful gifts of prognosis some men have! Here in this case, even in the condition described by the endorser, the prescience that could say that a patient with "profound coma, stertorous breathing," and threatened with "venous congestion of the brain," was "in a fair way to recover," is beyond not only my gifts, but those of medical authorities. I should be humiliated in finding myself so far below the gifts of the great practitioner who endorses the decision, if it were not for the good company I am in. The decision that the patient was "in a fair way to recover" was made in a few minutes after the victim was drawn from the pit, but Dr. Lyle and myself, with eight hours close watching of the case, and Dr. Lewis Rogers with seven hours observation, were not able to say, at the end even of that period, that this same patient was in "a fair way to recover." Throughout the hours in which the three physicians watched together, neither of us was able to bring himself to the positive conclusion that the case would recover, and when we parted at 2 o'clock, eight hours after the disaster, neither of us three was free from the apprehension of a fatal termination, though the patient slept "sweetly." And it is upon such judgments as I have described, that Dr. Rogers, Dr. Lyle, and myself are tried and condemned. But I submit this part of the case, just as it stands, to the judgment of the profession. Dr. Rogers is widely known as one of the first practitioners in Kentucky, and Dr. Lyle is one of the most promising of the young physicians in the city, and throughout the treatment of the case in question, there was neither clashing nor diversity of views among us. These gentlemen confirm the full truth of the picture I drew of the case in which they were concerned with me. And medical men can easily understand the declaration that we who attended the case, understood it quite as well as those who did not see it while we were treating it. There are few practitioners of medicine who have not met with just such quackish machinations and trickery as have been meted out to the physicians of this case; small cunning, mean inuendo, falsehood and injustice, and physicians every where can understand the meaning of these things. The blow was especially leveled at me, but each attempt upon me in relation to the treatment must equally affect Dr. Rogers and Dr. Lyle. That Dr. Bullitt made *deliberate* misrepresentations in this case, I have no kind of doubt. He speaks of the perversions of other men as though he were the paragon of honor, truth, and justice. Of his claims to the title we shall now make an estimate. In entering upon his noble and elevating course of conduct, a comparison of the treatment of the two cases, Dr. Bullitt says: "in the other case, the treatment of which was conducted by Dr. Bell." Here is a striking specimen of Dr. Bullitt's truthfulness. He knew that the treatment of the case was conducted by Dr. Lyle, Dr. Lewis Rogers, and myself, because, independently of any other source of information, the fact was prominently set forth in my report, and Dr. Bullitt professes to have read it. Dr. Bullitt wished to use Dr. Lyle as a witness against me, and pretends to be friendly to Dr. Rogers, and in this state of the case, he unscrupulously made the statement which I have quoted. This specimen of Dr. Bullitt's truthfulness will serve to show how much reliance may be placed on the rest of his statements.

Again; Dr. Bullitt asserts that there were marked differences in the results of the cases. I exceedingly dislike to touch anything of this kind, for Dr. Fry has no friend who feels prouder of the just and honorable reputation he won in the management of the German's case, than I do. He showed himself worthy of a place in the most honorable of all professions by his manly bearing, and his judicious course. And I feel sure that Dr. Fry is too correct in his gentlemanly deportment and professional knowledge, to set up a claim, that a difference of results, in cases treated by himself on the one hand, and on the other by Drs. Rogers and Lyle, must of necessity be caused by differences of treatment. Dr. Fry is physician enough to know that there are a great variety of circumstances that may make a difference in the result of cases, independently of any differences of treatment. In knowing this, Dr. Fry seems to have the advantage of Professor Bullitt.

But let us look at Dr. Bullitt's points of comparison of the treatment of the two cases:

1st. "There was a marked difference in regard to the recovery of the two patients."

This is true, but in a sense widely different from Dr. Bullitt's meaning. The patient of Dr. Rogers, Dr. Lyle, and myself was walking all over the city attending to his business, for many days before the German could leave his room.

2d. In the case of the German, "where depletion was resorted to as soon as the breathing and the circulation were re-established, and by other appropriate treatment, the coma and convulsions were soon overcome, and the patient was restored by the succeeding morning."

Well, in our case, as soon as Dr. Rogers, Dr. Lyle, and myself found our patient in a condition for bleeding, we bled him, and we were better judges of when the state of things came on that required the lancet than those who were not in attendance on the case. Dr. Adair Crawford covers the distressed Professor Bullitt precisely in his remarks on bloodletting in coma. Dr. Crawford says: "where a person is suddenly deprived of sense and motion, the practitioner is expected by the alarmed friends to employ instantly some active and efficient means of relief, and it is a very general rule with the profession to resort to the lancet, should there even be any doubt in the mind of the practitioner respecting the propriety of such a course, he may still be driven to adopt it, in deference to the opinions of the public, who ignorantly conclude that because bloodletting affords relief in some sudden fits, it is equally essential in all. If the patient recovers the cure is attributed to the venesection; and if he dies, it is hastily inferred that nothing could have saved him. If we turn, however to a calm consideration of the various and very opposite causes of comatose affections, it will appear a most obvious truth that nothing can be more contrary to the legitimate deductions of science, and more dangerous in practice than this indiscriminate and empirical use of the lancet. It has been seen that among the causes of coma, some are connected with morbid conditions of the substance of the brain, for the removal of which bloodletting is the only, if not a sovereign remedy; but that on the other hand coma is frequently to be ascribed to a deficiency or prostration of the nervous energy of the brain in which case it is evident that the substraction of any large quantity of the vital fluid can only have the effect of preventing the powers of the constitution from rallying, and of thus turning the scale against the patient,s recovery. We have little doubt that some of the recoveries from coma after bloodletting ought to be considered as truly escapes from death, rather than cures."

The character of Dr. Bullit's claims is well drawn by Dr. Crawford. I should place but little value upon Dr. Bullitt's opinion even if he had been present in the treatment of either case, but as he was snoring in bed, while Dr. Rogers, Dr. Lyle and myself were passing the night with our patient, endeavoring to save his life, Dr. Bullitt's opinion may be placed, where it belongs—at zero.

But to return. Dr. Bullitt would have his readers understand that the sufferings of our patient were protracted beyond those of the German. This shows either ignorance or recklessness, on the part of my great critic. Drs. Rogers and Lyle were invited by Dr. Fry at ten o'clock on the night of the disaster, to walk across the street to see the German—when they returned they thought our patient was in a better condition than the other. Various citizens were passing from one patient to the other up to a late hour of the night, and they uniformly described the relative conditions of the patients. Dr. Fry was over to see our patient after midnight, and Drs. Rogers and Lyle went over to see the German after that period, and during this time, being many hours after the German was bled, no claim was set up to any decided differences in the condition of the two cases. The relative position of the cases was freely spoken of among the attending physicians up to sometime after midnight, and no one claimed that the German was in a better condition than our patient. The comparisons made were uniformly in favor of our patient. For the truth of this statement I appeal to Drs. Rogers and Lyle. Dr. Lyle has had this read to him, and fully corroborates my statement. "The bleeding was delayed some hours," says our great practitioner. This is one truth, but Drs. Rogers and Lyle say it was not delayed beyond the period when it could be beneficial to the patient, and the benefit in our case was marked and decided at once.

3d. Dr. Bullitt says, "But in consequence of the delay of the bleeding, our patient continued to suffer for thirty six hours or more from coma, and was not really restored for several days." But according to Drs. Rogers, Lyle and Fry, representatives of the two cases, our patient was doing, at least as well up to 12 o'clock as the German, and no one of Dr. Bullitt's striking results of the difference in the time of bleeding was discoverable during the many hours that intervened between the time of bleeding the German, and the time we left our patient to repose on what we had done. In looking at Dr. Bullitt's extraordinary statements I wonder that he did not inform his readers that our patient died for want of bleeding, while his protogee was able to walk home at ten o'clock, and let his physicians go off to the enjoyment of a dance. These assertions would have been scarcely a greater remove from the truth than the statements Dr. Bullitt actually makes. But what are the facts? Dr. Bullitt says our patient was comatose for thirty-six hours, in consequence of the delay of the bleeding. But in my former report of the case I stated that our patient spake within ten hours of his attack, a fact well known to his family. There was not a symptom of coma in the case at that time, nor afterwards, as may be seen by referring to my report in the May number of this Journal, page 413, a report made at a time when I had no reason to suppose that any human being would fabricate such calumnies as those made by Dr. Bullitt. Instead of the existence of coma, for thirty-six hours, our patient walked down stairs, at his daughter's house, to a carriage, rode down home, got out of the carriage, walked up his lawn, then walked up stairs to his lounge, and walked up and down stairs as much as he pleased through the day, and all this without assistance, and this commenced within Dr. Bullitt's thirty-six hours of coma!

Drs. Rogers and Lyle, know that at our early visit next morning, within fourteen hours after the disaster, there was not a trace of coma, and we ceased using remedies. Dr. Rogers, Dr. Lyle, and myself were actively performing our professional duties while Dr. Bullitt was dreaming all the things with which he has adorned the pages of a Medical Journal. What a frightful nightmare the great physiologist must have had on that eventful night.

Dr. Bullitt says our patient "was not really restored for several days." Now nothing is more notorious in this city than the fact that our patient was walking all over the city attending to his large business, a number of days before the German was able to leave his room. Two weeks after the accident, Mr. Charles Taylor informed me that the German was still confined to his room, while our patient was confined but a single day! Nor is the German now, on the 25th of June, more than two months after the disaster, able to attempt to work, as Mr. Taylor, his employer, informed me he had ascertained by calling on him on the date I have given. So much for the marked results of the difference of treatment in the two cases! "Our friends" may well exclaim, save us from Dr. Bullitt's pen.

It would be hard to conceive of a more unscrupulous and reckless course than that which has marked Dr. Bullitt's officious intermeddling with this affair, and I do not envy him the position he has won by his folly and malice. What particular interest of science he has advanced, what great boon he has conferred on the healing art, what improvement he has made in the minds of his readers, by his conduct must be left to time to determine. In private practice, a physician who slanders a brother practitioner respecting the treatment of a case, who is reckless in his statements, and calumniates the best and purest conduct of his professional brethren, is justly regarded by all honorable physicians as degraded and infamous. Whether similar conduct on the part of the editor of a Medical Journal is not entitled to at least an equal measure of scorn and contempt, is left to the judgment of the profession. From such exemplars of professional courtesy, honor, decency and truth, as the author of the conduct I have described in this notice, I wish to be free during the remainder of my life.

I must add here that Dr. C. L. Lyle, authorizes me to use his name as corroborative testimony for the various statements I have made respecting the case we treated together.

### ADDENDA.

In making my original report of the case under review, my sole desire was to present to the profession a faithful and accurate description of one of the most interesting cases that ever passed under my observation. The report of medical facts of unusual interest is regarded by most medical men as an imperative duty, and under that influence alone, the report was made. It is not pretended that there was any allusion to the medical gentlemen engaged in the other case, consequently nothing offensive was done to them. I reported the facts as they passed under the observation of Dr. Lewis Rogers, Dr. Lyle and myself, and claimed nothing whatever for myself in the case. Dr. Rogers examined the report before it was published, and corroborated the facts that were presented. And in full view of the facts before him, with the printed report of the case in his hands, Dr. Bullitt, without cause or provocation, when, as he now says, our relations were friendly, selected me for a violent assault, when if his statements had been true, his attack would have affected Drs. Rogers and Lyle equally with myself. Dr. Bullitt claims that he undertook his work of detraction and abuse because he thought, at the time, that I had committed a breach of professional etiquette. Admitting that to be true, did it justify Dr. Bullitt in a wholesale misrepresentation of a case in private practice? When one medical editor gets mad at another, does that give him the privilege of descending to the walks of private practice, and of making statements to suit the phase of his anger? I appeal to the profession everywhere, nay to the moral sense of every one, whether any degree of anger can justify a medical Professor in slandering the private practice of a physician.

Dr. Bullitt is so sensible of the enormity of this outrage, that he does not attempt to defend himself. This constituted his chief offence, and he knew that he could not defend himself in any way. I may therefore take this as confession of judgment, and announce that Dr. Bullitt pleads guilty to the offence I charged upon him, that of falsely accusing a professional brother of what should have been charged only on the clearest and most indubitable proof. Dr. Bullitt admits that there were no unfriendly relations to cause him to make the charge he made, he knows that he can bring no kind of excuse to relieve himself from the charge of having made a false accusation, he cannot even plead the excuse that it was a blunder, or that he was misled by false information, for if any one had given him such information, he would himself have known that it was false. In the estimation of all professional men, I stand therefore, justified in the rebuke I administered to Dr. Bullitt, for his violation of the 9th commandment, and of the laws of the medical profession. There is not a medical man anywhere who would have submitted to such conduct, on the part of any professional brother, to say nothing of a medical editor and Professor, from whom people naturally expect proper conduct. *The grave and serious charge thus set forth in my response to Dr. Bullitt is not touched by him, simply because he knew his guilt and had no excuse for it.* I therefore wrap this garment of professional outrage around him, to cling to him the remainder of his life.

It is amusing to read Dr. Bullitt's coolness over my response to him. Because I would not submit patiently to his calumnies, but rebuked him as he deserved, he tries to imagine that I must have done something to him, that he knows nothing of. I think Dr. Bullitt has drawn on his imagination enough in this matter, without making that sweeping stretch, and he would consult the safety of his position, by sticking to facts.

In relation to the retraction of the word "deliberate" in connection with misrepresentation, of which Dr. Bullitt speaks, all that I have to say is, that feeling that it was a word unsuited to a scientific Journal, though its use was perfectly justifiable with the testimony before me, I intended to correct it in the August No. of the Journal. This intention I expressed to several of my friends, and among the rest to an intimate common friend of Dr. Bullitt and myself. After the reply appeared, this gentleman called upon me, as I understood him, for permission to mention my intention to Dr. Bullitt, and I gave it, and drew up a note for him, to guide him in his representation of the matter. But understanding that it was reported that this intended correction was the result of coercion, or that it had been drawn from me, I promptly sought the gentleman who conveyed the information to Dr. Bullitt, and informed him that I should make no correction of the word in the August No. of the Journal. And since Dr. Bullitt has not withdrawn his calumnious statement, but repeats it in another form, I shall make no change in my comment upon it.

I shall now endeavor to examine Dr. Bullitt's new researches in philosophy and science, and see on what foundations they rest. He swelled very largely in his first effusion about "the authorities," endeavored to create the impression that if I had been acquainted with them, I would have avoided my blunders, and he swaggered over his own acquaintance with them. I knew very well that Dr. Bullitt could not find one author, nor one authority in toxicology to sustain the absurdity he undertook to defend, viz: the propriety of making persons poisoned in sulphuretted hydrogen gas, continue to breathe the gas, and I said to him that I would surrender at discretion, if he would name one authority that sustained his notion. He does not produce one,—for the obvious reason that there is not one of them that hints at such a doctrine. Dr. Bullitt is the first author who has appeared in print in advocacy of the propriety or safety of suffering a victim to a poisonous gas, to breathe it one moment longer than can be avoided. Dr. Bullitt, in order to cover his retreat, quotes the authorities on thc *treatment of a case after it is removed from the gas*, *but no one of them advises any treatment in the gas.* When the authorities are speaking of the treatment of bodies after they are removed from the gas, Dr. Bullitt tears them from their context, and quotes them as though they were talking of treatment in a pit filled with the gas. Surely Dr. Bullitt does not suppose that he can impose on any intelligent mind with such sheer evasion and shuffling as this.

In asphyxia from drowning the first thing to be done is to remove the body from the fluid in which it had been drowned, and then the treatment commences. Now what would be thought of a Professor who would teach that the treatment might commence while the body was in the fluid, and quote the authorities on treatment to sustain his absurd notion, when no one of them ever thought of such a wild scheme? This is precisely Dr. Bullitt's condition.

There is not a solitary authority who does not lay down as the principle first to be acted upon, the removal of the body from the gas. Common sense would dictate that course, and science approves it. Raige-delorme, in the Dictionaire de Médécine, says, "*The first thing to be done is the removal of the body from the gas.*" Orfila, quoted by Dr. Bullitt in his former essay, says the same thing. Roget says: "The first step to be taken will naturally be to remove the body from the influence of the noxious gas, and expose it to a free and pure air." And in justification of the objections I urged, on account of the cold condition of the patient, Roget says: "*As the temperature of the body is generally raised above the natural standard, the affusion of cold water on the body may be employed with great advantage.*" If the increased temperature of the body then governs the use of the affusion, can it be claimed that the affusion is useful when the temperature is far below the natural standard? This principle laid down by Roget, shows that the treatment recommended by the authorities applies to cases removed immediately from the noxious gas. There is not one authority who advises any treatment of the body, while it is in the gas, and Dr. Bullitt should know this as well as I do. Neither Apjohn, Taylor, Christison, Parent Duchatelet, Guy, nor Levy, hints such a doctrine, and Dr. Bullitt's quotations might as well have been made from a report of the markets. I clearly set forth in my response to Dr. Bullitt that I did not object to the aspersion of cold water when the patient was in a pure atmosphere, but even this must be admitted with the proviso that the temperature of the body would permit such aspersion to be made. Dr. Bullitt's entire page and a half of authorities is devoted to wrenching them to the proof of a point that no one of them supports, while he has failed to produce one passage from any author on the point at issue. This, then, is an admission of the truth of my statement, when he loomed so largely upon "the anthorities." I said he could find no one who sustained him in saying that cold affusions of water should be made on persons while they were in a pit of sulphuretted hydrogen gas, and he has not produced one, nor can he do it. Until he does, he must submit to the charge of having talked largely and loosely upon a subject that he did not understand, and I shall soon produce other specimens of the same character. These are questions that no physiologist, except Dr. Bullitt, no toxicologist, has ever discussed. The first, the all-important matter is to get the body out of the gas, because each moment that it is permitted to remain in it diminishes the chances of resuscitation. The first great duty is to urge some resolute man, who can hold his breath forty or fifty seconds, the skin being partially protected by the clothing, to descend into the pit, and fasten a rope to the body. It would be as easy to procure ropes as buckets of water, and the body could be recovered at once.

Dr. Bullitt pretends to quote Guy in justification of his pet treatment. But Guy completely subverts Dr. Bullitt and sustains me. Instead of recommending water for the purification of the pit, Guy says: "*It is the custom at Paris to introduce a pan of burning coals into the impure air; this soon inflames the sulphuretted hydrogen, and purifies the air. When the burning coals are lowered into the gas, its presence is immediately recognized by the production of a luminous circle about the burning body.*" Here, then, is advice as to what is to be done with the pit, and as the plan commended by Guy is the one used by those who have more experience in such matters than any other people in the world, I am bound to believe that that is a much better management than the use of water. I have therefore clearly established my point by an authority who speaks to the question at issue. I said the victims could have been taken from the pit much sooner if pans of fire had been let down into the gas, and Guy commends that plan. Thus falls the ricketty structure erected, by Dr. Bullitt, with more labor than skill or knowledge. I felt convinced bv his first essay that he did not know what he was talking about, and his second attempt amply confirms it.

Dr. Bullitt never fails to misrepresent me whenever he has the least chance. Because I oppose the idea of forcing a person to breathe sulphuretted hydrogen gas,

Dr. Bullitt has it that I would do nothing, when I had already indicated that I should have tried to remove the bodies immediately into a pure atmosphere, in accordance with all the medical authorities, and this purification of the air, I should have attempted by letting down pans of fire into the gas, in conformity to the custom in Paris, where these matters are managed, not by vidanguers, but by scientific men. All this was too clearly presented to justify Dr. Bullitt's misrepresentations. His galvanic gyrations, then, about what I would have done, and what might have been the consequences, are thrown away. They are the wheezy struggles of a distressed logician mired in his own slough. The question which is most philosophical, to make a man breathe a gas so noxious that each inspiration is likely to destroy life, or to suffer him not to breathe at all, is not worthy of the attention of any medical man, and no one ever thought of starting any such folly until Dr. Bullitt committed his immortal production to the press. Taylor says: "a person who dies by sulphuretted hydrogen dies poisoned; as much so as if he had taken oxalic or hydrocyanic acid." If a body were found in which life was nearly extinct, with a bottle of hydrocyanic acid, in solution, at the mouth, the first thing to be done would be to remove the bottle. But if that could not be done, would any one excite the victim to inhale more of the hydrocyanic acid, by forcing him to breathe, or would it not be just as safe for him not to breathe, as to inspire such a noxious agent as hydrocyanic acid?

Dr. Bullitt very charitably says, in speaking of Michel Lévy's work on hygiene, "we doubt if Dr. Bell has ever seen a copy of it." Well, a copy of Lévy's work has been in my possession for sometime, and has been seen by numerous physicians. Thus falls that paltry slang.

Dr. Bullitt seems determined, in order to prove that I am dull for thinking of purifying a pit of sulphuretted hydrogen with fire, to upset the established principles and facts of chemistry. First, he will have it that the authorities do not support me, although I have given him Guy's authority for the plan; next, he has it that sulphuretted hydrogen does not burn but explodes. Let us see what a chemist says, and then it may be seen where the ignorance is, and who it is that does not understand chemistry. Brande says: "*Sulphuretted hydrogen extinguishes flame, but is itself inflammable in the contact of air, burning with a blue flame, and depositing during its slow combustion, a portion of its sulphur.—When mixed with excess of oxygen and inflamed it explodes.*"

Dr. Bullitt must see then, that my statement of the effect of fire on sulphuretted hydrogen gas, stands on higher authority than that of the vidangeurs of Paris. Guy and Brande both state the facts respecting the combustion of sulphuretted hydrogen, and Brande gives the chemical results. Now surely Guy and his American editor, Professor Charles A. Lee, and Brande understand this matter quite as well as Dr. Bullitt, and are not as likely to be effected with an optical delusion, as Dr. Bullitt is with a mental one on this point. That is certainly a rich piece of wriggling. Dr. Bullitt cannot state the most ordinary chemical truth without blundering. For instance, he says: "if the gas burnt it would be by explosion." This is not true. It burns in the atmosphere without any explosion, and it does not explode, even when there is "*an excess of oxygen*," unless a flame is introduced into it. But there is no explosion unless there is an excess of oxygen, and not even then when there is no flame. Come, Doctor, you must brush up your chemistry again.

I repeat, that I feel confident that if a man had been let down by a rope, he could have held his breath long enough to seize a body, or to fasten a rope to it, by which it could have been instantly brought into the open air. Christison records a case where a resolute man without any rope around him, went down into a pit filled with the poisonous gas, and recovered the body of a man who had fallen under the influence of the poison. Five minutes need not have been occupied in all the proceedings; getting ropes and all the rest of the means.

I have, therefore, shown that there is not one authority in toxicology that advises the use of water on a body that is lying in a pit of sulphuretted hydrogen gas—there is not one who hints that any means should be taken to make a person breathe in the gas, but each and every author quoted by Dr. Bullitt was advising the proper treatment for the victim when removed from the gas, and is torn from his purpose to sustain Dr. Bullitt. I have shown that Dr. Guy says, when speaking of the injurious effects of the gas upon animal life, that pans of fire "soon inflame the gas and purify the air." Dr. Bullitt's quotation from Duchatelet has nothing to do with the purification of a pit, but is descriptive of the method for cleansing a sewer, which can be opened in two places, whereby a current of air can be made to sweep out the gas. But this is not applicable to a pit, consequently it has nothing to do with the question before us.

Dr. Bullitt says that I contend that "inasmuch as sulphuretted hydrogen produces its poisonous effects when taken through the skin, it cannot produce asphyxia by acting on the nervous centers." I have contended for nothing of the kind. Dr. Bullitt loosely stated that the respiratory movements must be kept up, as though the gas could not destroy life, even when the lungs were exposed in a pure atmosphere, and the body was immersed in the poisonous gas. My argument was that the perfect freedom of the respiratory movements did not necessarily protect life if the skin weie exposed in any degree. We do not pretend to understand nervous centers as well as Dr. Bullitt does, but it would be strange that we could be ignorant of them after reading Dr. Bullitt's learned dissertation upon them. He underrates his teaching talent.

Having thus once more investigated Dr. Bullitt's scientific display, and found it wanting in all the elements of experience, authority, and truth, we dismiss him with the humble hope that he may turn his attention to the subjects of chemistry and physiology until he understands them well enough to avoid the blunders that are studded over his recent productions. He has contributed but little, as yet, to the science of medicine, although his opportunities have been abundant, and it is somewhat of a misfortune to him that he has acquired more notoriety by this foolish quarrel, which he started without cause or provocation, than by all his preceding efforts at scientific display.

I now turn to Dr. Bullitt's "incidental issues involving questions of fact."

First—in reference to Dr. Lyle. I stated on Dr. Lyle's own authority, that his name was used, in the attack on me, without any authority from him. He read over Dr. Raphael's description of the cases, and himself selected the items in the description, which he said were incorrect. Those were the points to which I objected, and Dr. Lyle said they were not present at the time they were said to be, nor for many hours after. Dr. Lyle then took my description, and read over each portion of it, and stated that each and every statement was perfectly correct. He read over everything mentioned in my article in reference to himself, and gave me liberty to use his name in corroboration of the statements. He then called and read over the article in proof slips, and authorized me to say that he approved every statement made in connection with his name. Before the Journal was published, he often talked to me on the matter, and never hinted that there was an error in any fact I had mentioned in connection with his name. Since the publication was made, he conversed with me, and was then firm in his corroboration of the facts, and he still stands firm in all these things. His testimony to the truth of each statement of my report, corroborated as it is by the evidence of Dr. Lewis Rogers places that report in an invulnerable position, challenging and defying attack from any quarter.

But Dr. Bullitt publishes a note from Dr. Lyle, of which the following is a copy:

DR. BULLITT:

*Dear Sir*—In reply to your note, I will state that I recollect having had a conversation with Drs. Raphael and Fry in your presence in reference to the cases of poisoning by sulphuretted hydrogen gas, and that I did agree with them in their account of the cases.

Very respectfully,

C. L. LYLE.

What particular influence is expected from this note, I do not know. Dr. Lyle says that "in *a conversation* with Drs. Raphael and Fry, in reference to the cases of poisoning by sulphuretted hydrogen gas, he agreed with them in their account of the cases." What does this amount to? What was the conversation? I do not use Dr. Lyle's name to contradict "a conversation," but to deny a publication, and Dr. Lyle says now, that he fully authorized everything I used his name to sustain. His note to Dr. Bullitt could be serviceable only in case we had the "conversation" in an accurate form before us, but we have not, and therefore we do not know in what matter, or what particular Dr. Lyle agreed with Drs.

Raphael and Fry. Dr. Lyle says himself, that he does not remember the purport of the conversation, further than that it was about the cases. Well, I have no doubt that I might agree with Drs. Raphael and Fry in a conversation on the cases, and might disagree about some special publication.

I wish this fact borne in mind then, that Dr. Lyle did not intend to contradict anything he had said in my report, as he assures me, consequently I have not a word to say against his note, as he wrote it. His note, and Dr. Bullitt's use of it are two different things. Dr. Lyle fully substantiated all my statements in connection with himself, and still sustains them in full.

I felt perfectly well satisfied that Dr. Lyle was not attempting to contradict the fact set forth in my response to Dr. Bullitt, that he corroborated my statements, but fearing that others might draw an improper conclusion, I addressed Dr. Lyle a note, which I give below, with his answer:

Dr. Lyle:

*Dear Sir*—Did I not have your consent to the use of your name as corroborative testimony to the truth of the statements of my report of the case of sulphuretted hydrogen gas poisoning, in which we were engaged as attending physicians?

Yours, respectfully,

August 6. T. S. Bell.

Dr. Bell:

*Dear Sir*—In reply to your note, you did.

Respectfully,

C. L. Lyle.

Dr. Bullitt manages to confuse every statement he attempts to make. In my reply to Dr. B. I said: "Drs. Rogers and Lyle were invited by Dr. Fry, at 10 o'clock, on the night of the disaster, etc., when they returned, they thought, etc." Dr. Fry called at my office one day and remarked that it had been suggested to him, that the phrase "when they returned," included him. He said he did not think so himself, but he thought he would ask me on the subject. I told him that it could not allude to him, because neither the grammatical construction of the sentence, nor the fact that he did not return with Drs. Rogers and Lyle, would point out such a meaning. Dr. Fry said nothing about the part relating to the 12 o'clock call, but even in that I did not represent him as saying anything to me. My impressions were derived from my two colleagues.

Dr. Bullitt says Dr. Rogers stands charged by Dr. B. with "the *unprofessional conduct* of passing back and forth several times between the two cases, making comparisons, &c." I cannot see how this would be unprofessional unless there was some attempt to disparage one of the cases. But why is Dr. Rogers alone selected for this slang? If he stands charged with this conduct, is he the only one implicated? Why, I repeat, select Dr. Rogers? But did Dr. Rogers say that by the mismanagement of one of the physicians, where both were acting together, a state of "coma" lasting "*thirty-six hours or more*" had been produced, when there did not happen to be any truth in the statement? I should regard such conduct as that flagrantly unprofessional, and should be pleased to hear Dr. Bullitt's opinion on the subject.

Dr. Bullitt certainly possesses a rare faculty for misrepresenting his opponent. Because in my original report I understated the results, and referred to the time when the patient was walking about freely, passing over the facts of Thursday, Dr. Bullitt says that either the first statement about Friday or the last one about Thursday, must be untrue. In order to make this out, he says: "If, as he reports in the first instance, his patient was not walking about *until* Friday, his statement in his reply that he was walking about within thirty-six hours of the accident is most certainly false." Here Dr. Bullitt makes a material addition to what I said, and by that addition makes a false issue, merely for the purpose of contradicting it. What must be thought of a medical man, a medical Professor, who can bring himself to resort to such miserable trickery? Did I say the patient was not walking about *until* Friday? Is there any such statement in my original report? It is Dr. Bullitt who slips in the word "until," and thus makes a statement for me, which I never made myself. And yet Dr. Bullitt holds that he is a truthful man and the pink of the profession. There is no kind of contradiction between my two statements as I made them—I was not engaged in my first report in making minute details of the convalescence, consequently I passed over the occurrences of Thursday. But when Dr. Bullitt made his marvellous statement about the "thirty-six hours *or more* of coma," the incidents of Thursday assumed an importance that they did not possess before, and I established by them, the fact that within Dr. Bullitt's "thirty-six hours *or more* of coma" the patient showed strong evidences of being free from coma. It is true the walking about on Friday might have been covered with Dr. Bullitt's statement, for his "thirty-six hours *or more*" might have alluded to six or nine months. Thus perishes this wordy trick.

Dr. Bullitt is exceedingly indignant because I did not state that the German's inability to work was on account of the burn on his foot. Dr Bullitt made a *limited* comparison of the treatment and results in the two cases. I extended the comparison, and met the matter fairly.—The application of the external warmth was a part of the treatment, and the result was a part of the case. In the German's case a cauterising-irritant was applied, and the foot dreadfully burned when the patient was as unconscious as a log. Dr. Bullitt is proud of the bleeding, we are proud of the temperate character of the applications we made to the feet of our patient. But why is Dr. Bullitt indignant at my reference to this burn? If C. J. B. Williams is an authority among medical men, I have no doubt that the hot brick had more to do with the rapid recovery from coma, claimed in the German's case, than any thing else that was done. The nervous centers, &c., were overpowered, and Dr. Williams says: "there seems to be another peculiar result from the applications of vehement heat, namely, a strong impression, or shock, on the nervous system, which in certain diseases is not without its salutary influences." Why should this burn excite so much indignation? When a man journeys to the stars, why kick the ladder down on which he climbed? The German's case is a verification of an old proverb, "the more haste, the less speed." In the general result we triumphed. We were censured without any cause, yet our patient got well without having his foot nearly burned off;—if we had met with such an accident in our treatment, it would have been loudly proclaimed everywhere, as a specimen of mismanagement. As a portion of the result of the poisoning the German got his foot so badly burned that he was not able to work for upwards of two months. In the other case the physicians personally superintended every application made to their patient, and he recovered from all the effects of his disaster more than two months before the German. Yet the German's case is Dr. Bullitt's pet.

I never made any allusion to the management of the other case either privately or publicly until I was forced to defend myself from a most unwarrantable attack. And when I did refer to it I stated the truth, and did not reach the enormity of "thirty-six hours *or more* of coma."

Having thus disposed of this imaginary case of "suppressio veri" or "suggestio falsi," I now call Dr. Bullitt to task on a real specimen of both in his own case. In his attack on me, which called forth my rejoinder, Dr. Bullitt represented that I was the only physician in a case in which two other physicians were in constant attendance, as he well knew. The entire treatment was conducted by Dr. Lewis Rogers, Dr. Lyle and myself. All three of us were equally responsible for the treatment. After making his statements say that I was the only physician, he represented, that by *my* delay of the bleeding, "the coma was made to last thirty-six hours *or more.*" The proof was palpable, before Dr. Bullitt at the time he made these statements, that Dr. Rogers, Dr. Lyle and myself were all engaged together in the treatment, and that the coma terminated in ten hours. I brought this home to the Doctor so forcibly that he consults his safety by making no allusion to it. He could not defend his enormity, his open, flagrant breach of professional decorum, etiquette, and morals, so he quietly slips away from his great outrage, without being able to look it in the face, upon finding it rather an unpleasant acquaintance. He takes one malicious bite at Dr. Rogers and Dr. Lyle as he retreats,—by calling me the Corypheus of the occasion, thus heaping his insults upon the heads of those gentlemen. If I was the Corypheus, what were Drs. Rogers and Lyle? Why are those gentlemen thus calumniated and libelled?

Things have come to a fine pass, indeed, when a man guilty of outrages that he cannot even defend has the effrontery to come before the community and lecture on truth, honor, professional courtesy, &c. I should think he would need a face of triple brass to sustain him on such an occasion.

For Dr. Bullitt's obscenities, calumnious inuendoes, and vulgarities I feel nothing but the most sovereign contempt. I am content to be his master in science, in medical philosophy, and in the truths of this case, and I

cheerfully surrender to him the whole territory of abuse, misrepresentation, and vulgarity, and his recent efforts show that he is no neophyte in such matters, whatever he may be in sciencc. The intelligent of the profession can easily draw the distinctions between us. He thinks his articles are not fit matter for a medical journal, and his medical brethren will agree with him, but why did he commence such matter in a medical journal? No one called him to the work of detraction, abuse and misrepresentation—he commenced the craft at his own option, and for his own purposes. We had been friends for many years, and there had been no cause on my part for our falling out. I never had an opportunity of doing Dr. Bullitt a service that was not cheerfully used for his benefit, yet in my whole life I never received as much injurious intention from any one, as was exhibited by Dr. Bullitt, as he says himself, while he was friendly. It is hard to determine which are the queerest, Dr. Bullitt's views of the offices of friendship, or of science. He thinks that one may be used for calumniating and misrepresenting a friend, and that the other may be employed in distorting, perverting and misusing scientific authorities, to make them suit any special present object.

But there is one truth that must strike the attention of every reader. If Dr. Bullitt's spiteful and malicious description of me in his sublime peroration has any truth in it, how does the Doctor stand in view of the declaration in his exordium—that we had always been friendly until this outbreak came? Dr. Bullitt himself, either does not believe a word of that tirade, or he has singular qualities for friendly feelings. I hold myself above and beyond the reach of such stuff. I introduced no allusions to Dr. Bullitt, extraneous to the issues he presented in his attack, and the introduction of them by Dr. Bullitt shows how hard pressed he was. I needed no such aid, for I

had the truth, and I verily believe that that is stronger than any species of abuse.

In reference to Dr. Builitt's projected series of medical papers in review of my medical essays, I have nothing more to say, than that I shall be pleased to see him even from spitefulness engaged in doing something for the progress of his profession. The Doctor will find me ready whenever he commences his trade of reviewer. If he shall be as brilliant in other things as he is in fetid gases, and as accurate in stating facts, and quoting from an opponent as he has proved himself in his recent two attempts, he will not be very formidable.

